# Melatonin Is a Feasible, Safe, and Acceptable Intervention in Doctors and Nurses Working Nightshifts: The MIDNIGHT Trial

**DOI:** 10.3389/fpsyt.2020.00872

**Published:** 2020-08-27

**Authors:** Bensita M. V. J. Thottakam, Nigel R. Webster, Lee Allen, Malachy O. Columb, Helen F. Galley

**Affiliations:** ^1^ Institute of Medical Sciences, University of Aberdeen, Aberdeen, United Kingdom; ^2^ Intensive Care Unit, Aberdeen Royal Infirmary, Aberdeen, United Kingdom; ^3^ Anaesthesia and Intensive Care Medicine, Manchester University Hospitals NHS Foundation Trust, Wythenshawe, United Kingdom

**Keywords:** melatonin, randomized controlled trial, shift work, sleep, healthcare workers

## Abstract

Nightshift working is associated with sleep deprivation, fatigue and attention/concentration deficits which, in healthcare workers, may impact on patient safety. Clinical staff in the UK routinely work several 12 h nightshifts in a row at about 1–3 month intervals. We investigated the feasibility and acceptability of a crossover trial of melatonin administration in clinical staff working nightshifts with an exploration of effects on sleep measures and attention/concentration tasks. This was a pilot, double-blinded, randomized, placebo-controlled crossover feasibility trial in doctors and nurses working 3 consecutive nightshifts at a tertiary referral hospital in the UK. Twenty five male and female subjects were randomized to receive either 6mg Circadin™ slow release melatonin or placebo before sleep after each consecutive nightshift, followed by a washout period, before crossing over to the other experimental arm. We used actigraphy for objective assessment of sleep parameters. The trial design was feasible and acceptable to participants with negligible side effects, but elevated melatonin levels were prolonged during the active arm (P=0.016). Double digit addition testing, a concentration/attention task, improved with melatonin treatment (P<0.0001). Lapses of vigilance or judgement while doctors or nurses are working nightshifts could impact on patient safety and melatonin may be a useful intervention. This study supports the conclusion that a larger definitive trial of this design is both feasible and safe.

**Clinical Trial Registration:** identifier ISRCTN15529655. https://www.isrctn.com/

## Introduction 

Healthcare workers are reported to typically sleep only 4–7 h between nightshifts, associated with sleep deprivation, fatigue, and performance deficit (1–4). There are increased risks of myocardial infarction (5), type II diabetes (2), and rheumatoid arthritis (6) and there are also more occupational injuries during nightshift working (7, 8).

Junior doctors in the UK routinely work three or four 12 h nightshifts at intervals of around 1–3 months and nurses may work up to 7 nightshifts in a row. Similar patterns of either 8 h or 12 h are common in hospitals in the USA (9, 10) and elsewhere in Europe (3) but nightshifts can last up to 16 h in Japan (11).

Lapses of vigilance or judgement due to sleep deprivation while clinical staff are working nightshifts could impact on patient safety. So-called jet lag has similar effects to shift working and systematic reviews suggest there is evidence to support the use of melatonin. The only licensed form of melatonin in the UK is Circadin™, a modified release formulation, licensed for treating primary insomnia in older adults. The feasibility, safety and acceptability of its use for clinical staff working nightshifts is not known. We therefore undertook a pilot, double-blinded, randomized, placebo-controlled, 2x2 crossover feasibility trial of Circadin in doctors and nurses working real life nightshifts at a tertiary referral hospital in the UK. We also undertook an initial exploration of efficacy in terms of sleep parameters and effects on concentration and attention tasks, to inform a subsequent definitive trial.

## Methods

The Medicines and Healthcare Regulatory Authority (MHRA) ruled that this was not a Clinical Trial of an Investigational Product (CTIMP) and a Clinical Trial Authorization was therefore not required. Following ethical committee (North of Scotland Research Ethics Service, reference 16/NS/0010) and NHS Grampian management (reference 2016AN001) approvals, expressions of interest in taking part in the study were invited *via* emails sent to all acute care nurses and trainee doctors throughout NHS Grampian, which comprises Aberdeen Royal Infirmary, Royal Aberdeen Children’s Hospital and Aberdeen Maternity Hospital, plus outlying hospitals. Any doctor or nurse who worked 12 h shifts of at least 3 nights in a row was potentially eligible, as long as they worked during the entire shift with no opportunity to nap, and each set of shifts was about a month apart. Shift start times were 19:30 or 20:00 and were of 12 h duration for all subjects. Nobody finished earlier and nobody worked more than 15 min longer.

Subjects were excluded if they were pregnant or breastfeeding, had a pre-existing chronic health condition and/or took any regular medication other than oral contraceptives, had a body mass index (BMI) of more than 30 Kg/m^2^ or were smokers. To reduce the possibility of recruiting any female participant who may become pregnant, all women were asked confirm they were either on reliable contraception, had been sterilized, were post-menopausal or declared themselves to be sexually inactive, and so were not likely to become pregnant.

Confirmation of participants’ eligibility and clinical oversight was provided by Dr Allen and Professor Webster, both Consultants in Intensive Care Medicine. The study was monitored by NHS Grampian and a Data and Safety Monitoring Committee was appointed. After written informed consent, subjects were randomized to receive either 6mg Circadin™ (Flynn Pharmaceuticals Ltd.) or a placebo of identical appearance, before sleep during their first series of three shifts and the opposite for the second series of three shifts. The randomization schedule was provided to the Clinical Trials Pharmacy in advance by an independent statistician not involved in the trial, in blocks of 8 to further minimize any seasonal effects. Circadin is a modified release formulation with a blood concentration profile resembling that of endogenous melatonin. Both Circadin and the placebo were provided by Flynn Pharma and were repackaged, labeled and dispensed by the Clinical Trials Pharmacy at Aberdeen Royal Infirmary who were also responsible for drug accountability. The study was sponsored jointly by the University of Aberdeen and NHS Grampian. Flynn Pharma had no input to study design or conduct.

**Figure 1:**
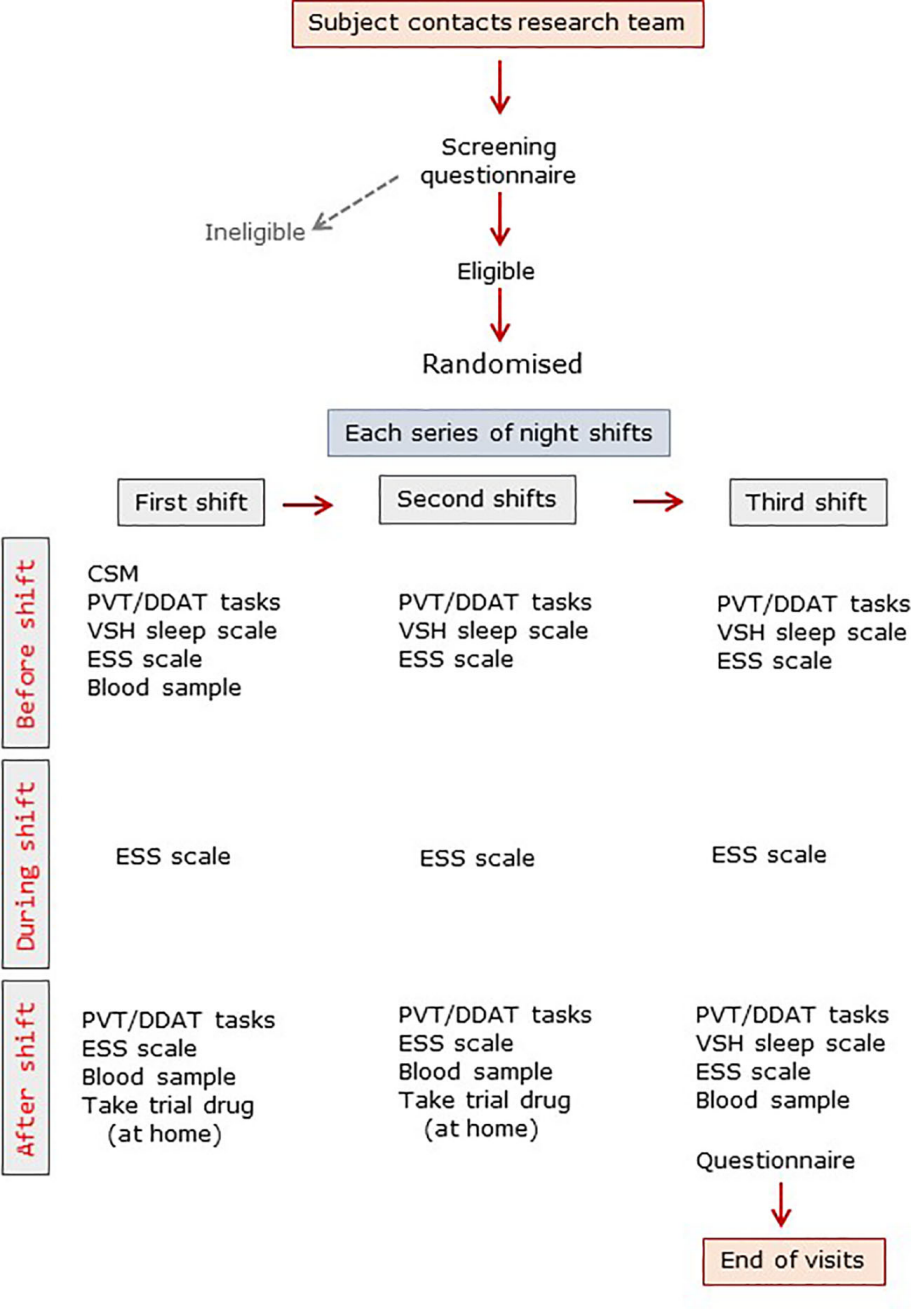
Trial visit summary. ESS, Epworth sleepiness score; CSM, composite scale of morningness; DDAT, double digit addition task; PVT, psychomotor vigilance task; VSH, Verran-Snyder-Halpern sleep scale.

The trial design is summarized in **Figure 1**. Subjects were allowed to familiarize themselves with the various tasks beforehand. Subjects completed the Composite Score of Morningness (CSM) (12) and, immediately before the first night shift they completed the Verran Snyder Halpern (VSH) sleep scale, a validated assessment tool which uses 100mm visual analogue scales to record self-reported sleep disturbance, efficiency and supplementation in the previous 24 h period (13). A psychomotor vigilance task (PVT) was undertaken on a laptop computer with a Razer Abyssuss™ high performance gaming mouse (Amazon.co.uk). The test requires sustained attention and measures reaction time to a visual stimulus (14). Subjects also completed a timed double-digit addition task (DDAT) where a random sheet from 25 different sheets of 20 double-digit sums was provided and subjects were given 30 s to complete as many as possible as accurately as possible (15). No subject received the same DDAT sheet more than once. Finally, subjects completed the Epworth Sleepiness Scale (ESS), which records self-reported situational propensity to fall asleep (16). Blood samples were taken before and after shifts (see below). During the nightshift subjects worked all night and were not able to nap. They were sent automated text messages at 04:00 to remind them to complete the ESS at that time.

The following morning, immediately after the shift, participants completed the PVT, DDAT, VSH and ESS assessments again. They were given nine x 2 mg Circadin or placebo tablets and asked to take 3 tablets (6 mg) with a snack at home after each shift, and to go to bed within 2h of taking the tablets. The time of taking the tablets and the time of going to bed/getting up were self-reported. Subjects wore sunglasses on the way home to minimize light exposure and were given an Actiwatch™ (Phillips Spectrum PRO, Linton Instrumentation Ltd., Diss, Norfolk, UK) to wear. Actiware™ software (Philips Respironics, V6.0) enables analysis, management and export of objective activity data, providing times of going to bed and getting up, duration of time in bed, time actually asleep, sleep latency, and minutes awake after sleep onset (WASO); it concurs with polysomnography (17). Participants only wore the Actiwatch at home as watches cannot be worn during clinical time. We asked participants after every shift about napping; all staff reported that they had not napped. At the start of the next shift all measures were repeated and the Actiwatch data downloaded. On the fourth evening final assessments were undertaken and the medication bottle was collected. Participants were asked daily about adverse effects and were given a post-participation questionnaire which they completed anonymously and returned in a sealed envelope; these were not opened until recruitment was complete.

### Biochemical Assays

Blood samples were collected into clot activator tubes and centrifuged within 2 h of collection for 10 min at 2,300g and the serum stored at -80°C. Melatonin was measured using liquid chromatography-tandem mass spectrometry, reported in full elsewhere (18). The assay monitors levels after exogenous melatonin administration with a lower limit of quantification of 50 pg/ml. Blinding was maintained throughout. Biochemistry (urea and electrolytes and liver function tests) and hematology (full differential leukocyte counts and hemoglobin) tests were undertaken using standard methods in our hospital laboratories.

### Statistical Analysis

The primary aim of this pilot study was feasibility and acceptability of the trial design, and safety, with an exploration of effectiveness in a real-life situation. Our primary outcome measure was 25 subjects completing both cross-over arms; this number was considered to be sufficient for a pilot study using a crossover design. Secondary aims were to assess melatonin levels and to explore effects of melatonin and nightshifts on sleep measures and attention/concentration tasks. Data are presented as median and full range and were analyzed using mixed models with maximum likelihood estimation for repeated measures in a crossover design. This included tests for treatment (melatonin), period and treatment by period interactions (sequence or carryover effects). Bonferroni multiple comparison corrections were applied to keep the overall type 1 error rate at <5%. Non-parametric analysis was used as appropriate. Significance was defined as P<0.05 (two-sided). Analyses were conducted using Number Cruncher Statistical Systems 12 (NCSS), NCSS Inc., Kaysville, UT, USA, StatXact 9, Cytel Inc., Cambridge, MA, USA and StatsDirect 3, StatsDirect Ltd., Cambridge, UK.

## Results

### Recruitment, Feasibility, and Participant Feedback

**Figure 2:**
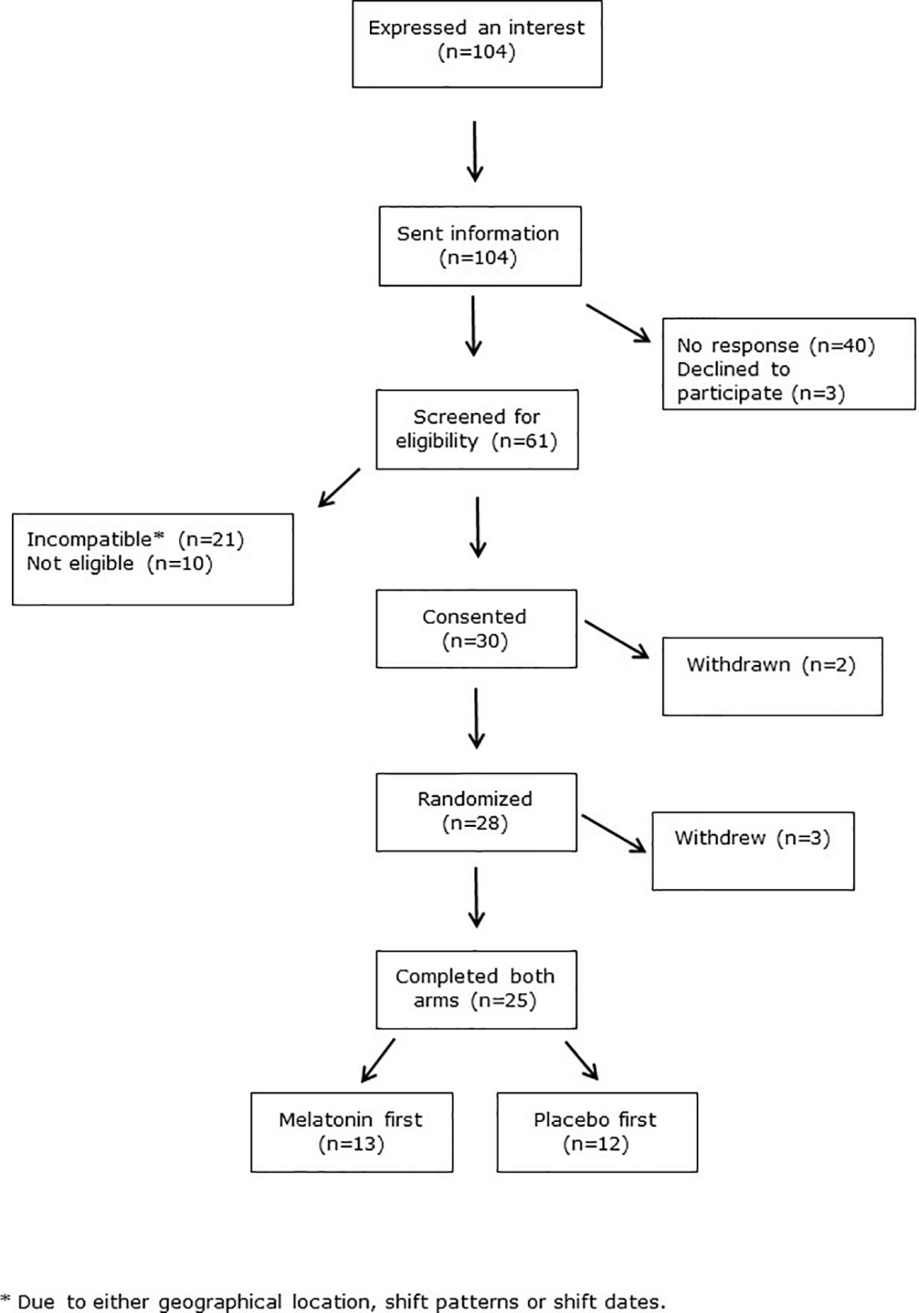
CONSORT diagram.

Crossover trials are renowned for higher attrition rates than other trial designs. We therefore allowed for 30% dropout, and aimed to recruit 32 subjects to achieve our primary outcome measure of 25 subjects completing both crossover arms. However our dropout rate was lower than anticipated such that this was achieved after recruiting 30 subjects. The CONSORT flow diagram in **Figure 2** shows recruitment data. There were 104 initial expressions of interest but 40 people did not respond after receiving further information. Three people declined to take part. Ten subjects were ineligible, due to smoking, BMI>30 Kg/m^2^ or chronic medication use. Twenty-one subjects were eligible but unable to take part due to incompatibility of shift patterns or dates, or geographical location. Two subjects were withdrawn after consent but before randomization, as they were subsequently found to meet the exclusion criteria. Three more withdrew after randomization, one for personal reasons, one due to acute ill-health unrelated to the trial, and one left the geographical area. The median [range] duration between each shift series (=periods) was similar between those who had placebo and those who had melatonin first (6.7 [3.6–14.4] and 7.7 [3.9–13.0] weeks, respectively, P=0.43, **Table 1**).

**Table 1:** Characteristics of trial participants.

	**Placebo first (n = 13)**	**Melatonin first (n = 12)**
**Age (years)**	28 [24–56]	27 [24–56]
**Sex (male:female)**	4:9	4:8
**BMI (kg m^-2^)**	24.3 [18.1–30.0]	23.0 [19.3–27.3]
**CSM score**	31 [20–44]	42 [23–50]
**Weeks between treatment arms**	6.7 [3.6–14.4]	7.7 [3.9–13.0]

Data are median [full range].

BMI, body mass index; CSM, composite scale of morningness.

The characteristics of subjects and CSM scores for each randomized sequence were similar (**Table 1**). All subjects completed all scheduled tasks and there were no protocol violations, no serious adverse events and very few adverse events. Three subjects reported headaches, revealed later to be one during the placebo arm and 2 during the melatonin arm, although one of the latter had not yet taken any melatonin. Two people reported “strange” dreams, one during placebo and one during melatonin. One person reported being “excessively drowsy” after taking trial medication (later revealed to be melatonin).

Anonymous post-trial surveys were returned by 24/25 (96%) participants (**Table 2**). Five-point Likert scales (strongly agree/agree/ambivalent/disagree/strongly disagree) recorded responses to statements. The final question asked if subjects would take melatonin when working nightshifts if it was available (**Table 2**). Overall, 88% strongly agreed/agreed that the trial was well organized and 100% strongly agreed/agreed that communication was good. Overall 54% strongly disagreed/disagreed that they were inconvenienced by taking part, 5 (20%) people were ambivalent and just one person felt inconvenienced. Eleven (46%) agreed/strongly agreed they would take melatonin if it were available, 12 (50%) were unsure if they would or not and one person said they would not take it.

**Table 2:** Post-trial questionnaire responses.

Question	Strongly agree/agree	Ambivalent		Disagree/strongly agree
The trial was well organised	21	3		0
Communication was good	24	0		0
Taking part was not too onerous	19	4		1
There were too many tasks	3	3		18
There were too many blood samples	8	8		8
There were too many questionnaires	1	5		17
Taking part inconvenienced me	1	5		13
I had side effects from taking part	1	3		20
I would take melatonin if it was available	11	12		1
I think I know if I had melatonin or placebo first	**YES** 11		**NO** 13	

### Blood Analyses

There were no differences in hematology and biochemical data at baseline and although there were some statistically significant changes over the course of the study, these were considered to be clinically irrelevant (**Supplementary Table S1**).

**Figure 3:**
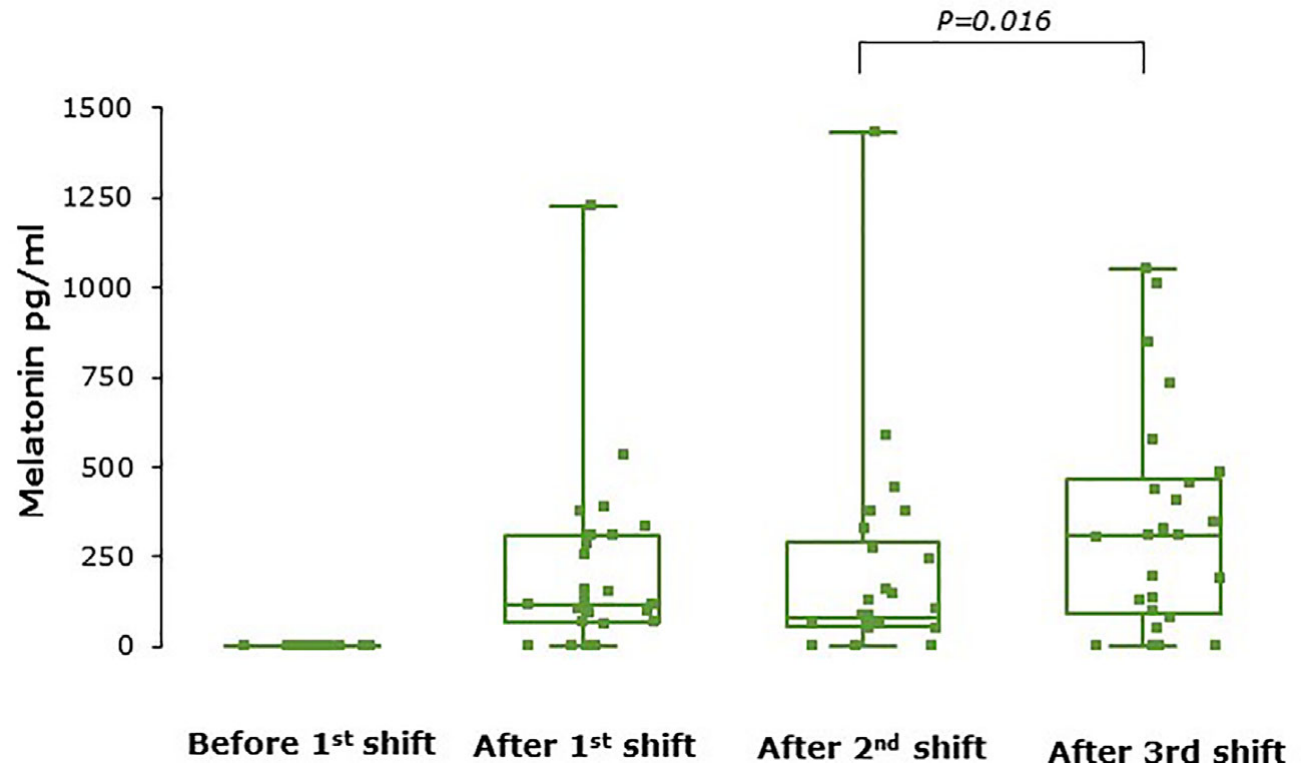
Serum melatonin concentrations at baseline and after each shift during the melatonin arm (n = 25). Levels increased significantly over the series of shifts (P = 0.01 overall) and were higher after shift 3 than shift 2 (P = 0.016). Melatonin levels were not detectable at baseline in any participant. Box and whisker plots show median, interquartile, and full range with individual data points overlaid (n = 25).

Serum melatonin concentrations were below the lower limit of quantification (50pg/ml) in all samples during placebo shifts and in all baseline samples (i.e., before the first shift for both treatment arms). **Figure 3** shows serum melatonin levels after each shift during the melatonin treatment periods only. Melatonin levels were very variable between individuals such that some participants had circulating melatonin levels below the lower limit of detection during the melatonin treatment period while others had very high levels. There was a significant effect of shift on melatonin levels (P=0.01) and levels were significantly higher after shift 3 than shift 2 (P=0.016). No unused tablets were returned.

### Sleep

**Figure 4:**
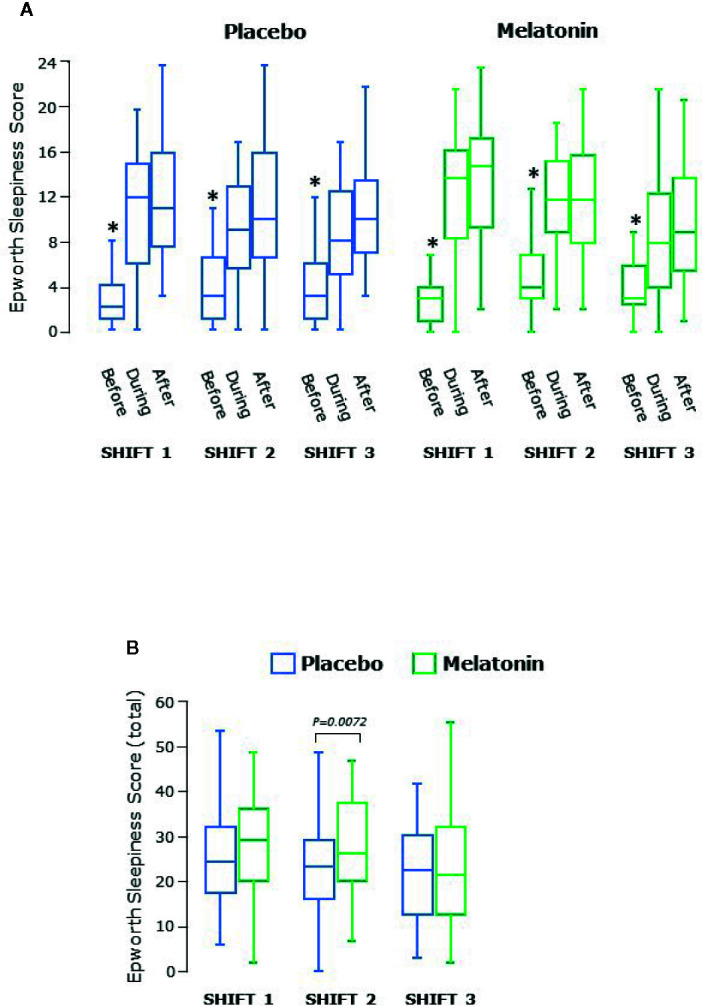
**(A)** Epworth sleepiness score before, during and after each series of shifts. ESS was significantly lower before than during and after each shift (P<0.0001 overall) and higher after the shift than during the shift (P = 0.0045 overall). *significantly lower than during or after shift, p<0.05. **(B)** Total Epworth sleepiness score for each shift. Scores decreased significantly over the three shifts (P = 0.0026 overall). Box and whisker plots show median, interquartile, and full range (n = 25).

ESS is a self-reported score assessing situational propensity for dozing or “sleepiness”. There was pronounced variation between participants but it is clear that ESS was lower before every shift than during and after each shift during both placebo and melatonin treatment arms (both P<0.0001 overall, **Figure 4A**). There was also a shift effect (P=0.0026) such that that ESS was lower throughout shift 3 than shifts 1 and 2 during both treatment periods (**Figure 4B**). In addition, overall there was a significant treatment effect with increased ESS during melatonin treatment due to higher ESS scores overall during shift 2 (p=0.0072, **Figure 4B**).

Sleep latency, duration and WASO were assessed objectively using Actigraphy, and subjective sleep disturbance and efficiency were assessed using the VSH scale (**Table 3**). Latency (the time taken to fall asleep) was variable but in all cases less than 16mins and was not affected by nightshift working. There were no significant effects of treatment found. Sleep duration was a median of 5–6 h but some participants slept for as little as 2 h and some as long as 8h. Duration of sleep decreased over the shift series in both treatment periods (P<0.0001, **Table 3**) with over an hour less sleep after shift 3 than after shift 1. However no significant effects of melatonin treatment were seen. The amount of time participants spent awake after going to sleep (WASO) was a median of about 30 min and decreased by 10 min over both shift series (P=0.012 **Table 3**).

**Table 3:** Sleep measures.

Measure	Treatment arm/shift						Analysis	
	Placebo			Melatonin			Shift	Treatment
	1	2	3	1	2	3		
**Latency** (min)	**4.0** [1.1–9.0]	**8.5** [0.9–14.6]	**7.3** [0.0–15.8]	**2.5** [0.0–6.9]	**4.5** [0.0–9.0]	**5.0** [0.0–13.0]	P=0.26	P=0.17
**Duration** (hr)	**6.46** [3.95–8.30]	**5.79** [2.00–8.43]	**5.28** [2.00–7.88]	**6.32** [3.58–8.32]	**6.57** [5.00–8.32]	**5.33** [1.68–8.23]	P<0.0001	P=0.19
**WASO** (min)	**28.3** [13.0–78.5]	**30.5** [13.5–65.5]	**25.0** [1.0–68.5]	**28.0** [11.0–82.0]	**30.5** [12.0–83.5]	**21.5** [5.0–61.0]	P=0.12	P=0.11
**Disturbance** (score)	**192** [0–447]	**198** [0–449]	**160** [11–428]	**196** [30–579]	**185** [0–609]	**114** [2–643]	P=0.026	P=0.77
**Efficiency** (score)	**390** [199–489]	**390** [160–487]	**363** [122–474]	**356** [177–532]	**382** [100–487]	**321** [197–480]	P=0.066	P=0.85

Median [full range]. WASO, wake after sleep onset. The median values are in bold.

The maximum possible score for sleep disturbance recorded using the VSH scale is 700; a lower score indicates less disturbance. Disturbance scores were very variable and in some participants were higher than we found previously in healthy young men (18). Disturbance score decreased over the shifts (P=0.031) and was most marked after shift 3 during melatonin treatment although this did not reach significance (**Table 3**). Sleep efficiency recorded using the VSH scale has a maximum score of 500; values here were similar to what we have seen in healthy young men (18) (**Table 3**).

### Concentration and Attention Tasks

**Figure 5:**
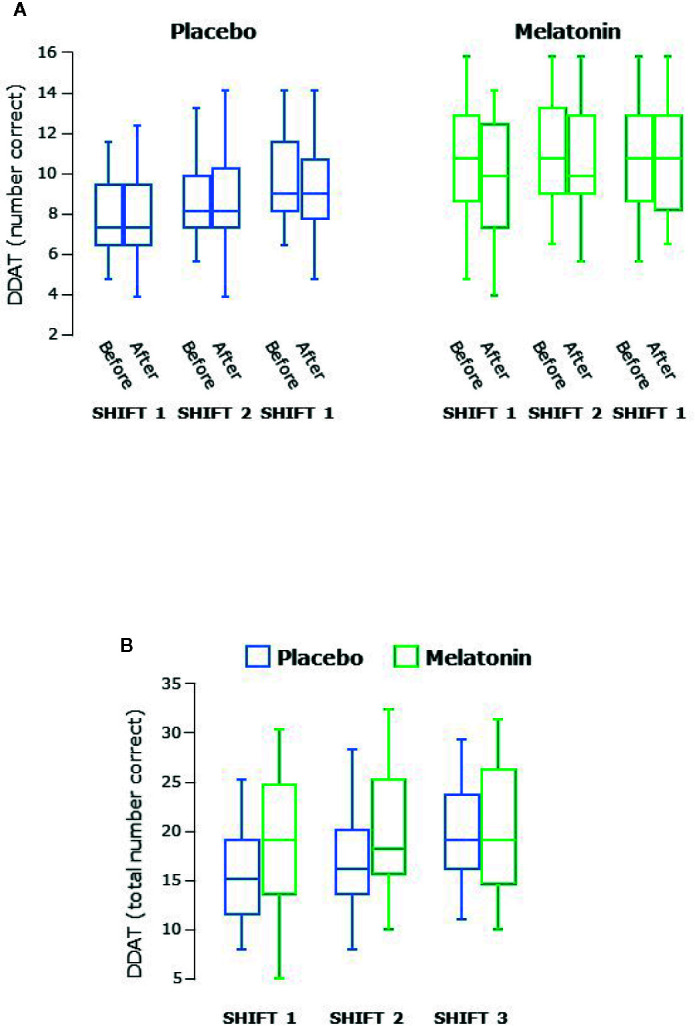
**(A)** Double digit addition testing (DDAT) before and after each series of shifts. **(B)** Total number of correct answers in DDAT for each shift. Scores increased significantly over the three shifts during both treatment arms (P < 0.0001 overall) and were higher during melatonin treatment (P < 0.0001 overall). Box and whisker plots show median, interquartile, and full range (n = 25).

Concentration and attention was assessed by double digit addition and reaction time testing. The range of the number of sums correctly answered in the DDAT test varied from 2 to 16. There was an increase in the number correct over both series of shifts (P<0.0001 overall, **Figure 5A**) and scores were higher during melatonin treatment (P<0.0001, **Figure 5B**). There were no differences between males and females.

**Figure 6:**
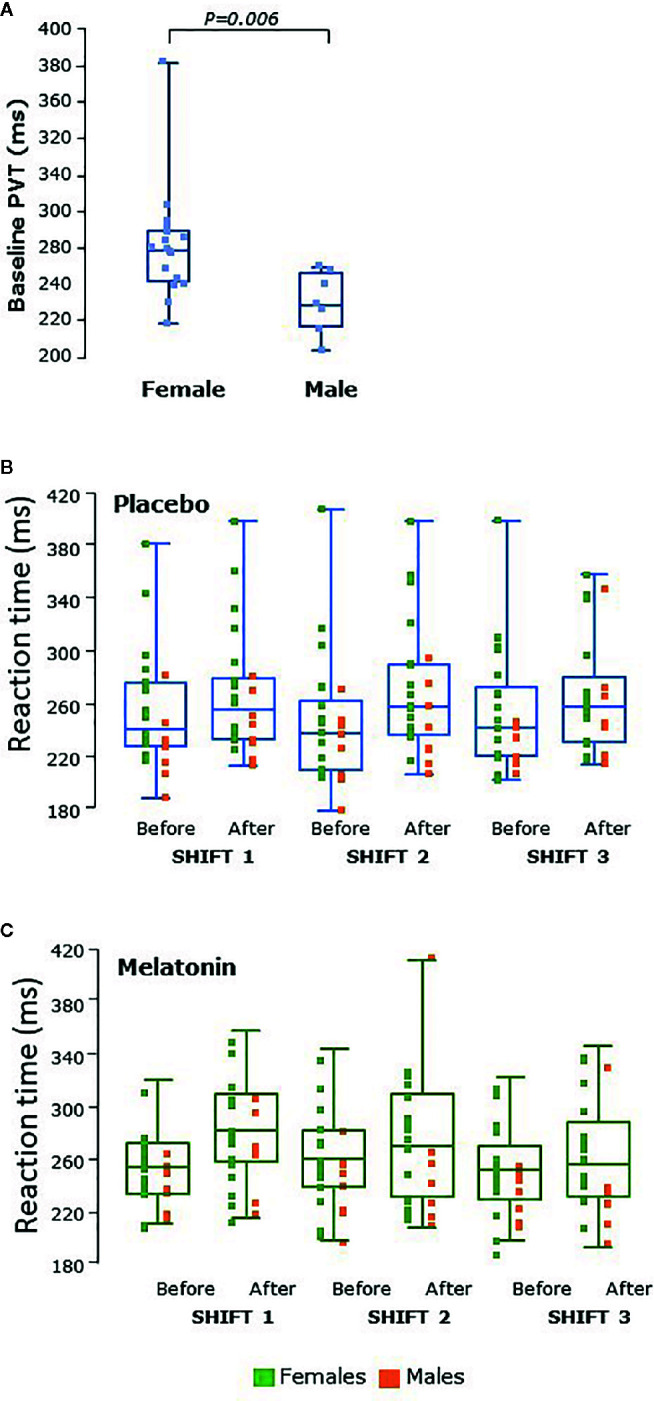
**(A)** Psychomotor vigilance testing (PVT) as reaction time in male (n = 8) and female (n = 17) participants at baseline, before the first shift regardless of treatment arm. Reaction times were significantly faster in males than females at baseline (P = 0.006). **(B)** PVT tests before and after placebo shifts and **(C)** PVT before and after melatonin shifts. Female participants’ data points are shown in green and male participants’ in orange. Reaction times were faster in males than females during all shifts. Reaction times were significantly higher after shifts (P = 0.0007) in both males and females than before. Box and whisker plots show median, interquartile, and full range with individual raw data points overlaid.

Reaction times varied between 200 and 400 ms and male participants had significantly faster PVT reaction times than females at baseline, before the first shift (P<0.006, **Figure 6A**). Reaction times were slower after shifts than before in both sexes (P=0.0007, **Figures 6B, C**). There were very few PVT lapses (>500 ms, data not shown) and no significant difference between melatonin and placebo treatment periods was seen.

## Discussion

We report here that this rigorous pilot Phase II feasibility trial of Circadin compared to placebo in doctors and nurses working real life nightshifts was feasible, safe, and acceptable to participants. All subjects completed both arms of the study with no protocol violations. There were negligible side-effects with no wellbeing or safety concerns, and compliance with the protocol was excellent. Although this was a pilot study and any effects seen were secondary outcome measures, working nightshifts significantly affected sleep disturbance, duration, and time spent awake after sleep onset and subjective sleepiness was poorer during shifts. Circulating melatonin levels were very variable between individuals and were undetectable in some but remained high in other subjects during Circadin treatment even 8 h after taking the tablets. Attention and concentration tasks were significantly impacted by nightshift working and we found some differences between males and females. Melatonin treatment was associated with improved double-digit addition task scores.

Previous studies have shown that working nightshifts impacts on health generally and on functional performance during the shift itself, both in simulation studies and real-life workers (1, 2, 5, 19, 20). Here we show the study design for a trial of administration of melatonin was feasible and acceptable to participants in real-life working situations for clinical staff in a hospital environment. The intervention was well tolerated and not associated with adverse events, thus raising no safety concerns. We found no differences between those receiving melatonin first and those receiving placebo first. Although there was a possibility that giving melatonin during nightshift working to help daytime sleep may have meant there were issues on return to dayshifts, nothing was reported by any participant. Notably, none of the participants who were randomized to receive melatonin first reported sleep problems on return to day shifts after the first shift series. However, we concede that this was not the focus of our study and we did not formally assess sleep after return to dayshift working. We did not restrict normal activities, nor dictate participants’ sleeping habits prior to starting night shifts, such as napping during the day, staying up late, or all night. There was no restriction on exercise during the study, or on caffeine/food intake or timing of daytime sleep, although we did ban alcohol consumption in the 24 h before the shifts and during the entirety of the two series of shifts. The primary study aim was to determine feasibility and safety but with robust multivariate cross-over analysis to comprehensively explore any influence of nightshift working on sleep parameters and attention/concentration tasks including analysis of the impact of randomization sequence, shift, period (i.e., shift series) and melatonin treatment to inform a subsequent definitive trial. No previous studies have attempted to dissect temporal effects which may compound interpretation of the effectiveness of an intervention in this situation in this participant population.

Crossover trial designs use longitudinal measurements which minimize seasonal effects by randomizing to treatment sequences. Confounding variables are reduced as each subject acts as their own control. Crossover designs are statistically efficient and require far fewer participants than other repeated measures designs. As such, this design was appropriate here. The main disadvantage of the design is carry-over. However we allowed an adequate washout period between periods -this is usually recommended to be 5 x the half-life of the drug. The Summary of Product Characteristics for Circadin reports that the time to maximum circulating levels in the non-fasted state after a 2 mg dose is around 3 h, with a half-life of approximately 4h and complete elimination by 12 h. Our median time between treatment arms was around 7 weeks. In addition serum melatonin was not detectable before each treatment arm in any participant. Although cross-over designs often have a high dropout rate due to participants being in the trial for longer than other trial designs, this was not the case here, possibly due to the participant demographic.

We have described the assay for melatonin previously (18); slight adjustments were made to improve the lower limit of quantification given the lower dose of melatonin used here. The method is designed to analyze melatonin levels after exogenous administration and is not sensitive enough to detect endogenous levels; it was therefore unsurprising that melatonin was undetectable in baseline samples or during placebo treatment. Circadin is a modified release formulation of melatonin and we found that circulating levels were highly variable during treatment and in some cases undetectable in a few individuals, yet markedly elevated in other individuals even 8–10 h after they had taken Circadin. Most previous interventional studies do not report melatonin levels and there is no consensus for an appropriate dose. We and others have reported considerable inter-individual variation in melatonin levels after administration of immediate release melatonin (18, 21) thought to be due to genetic polymorphisms of liver enzymes responsible for first pass metabolism (22). We did find that prolonged elevation in melatonin levels and possible accumulation seen here was not associated with any functional deficit. The increasing levels over the shifts during melatonin dosing suggests delayed clearance by those with a slow metabolizing phenotype, and/or accumulation due perhaps to the dose used. Circadin treatment appears to be very safe with negligible side effects in this population of healthy subjects, as we have reported previously using an immediate release formulation, even at doses of 100mg (18). A recent trial of 2 mg Circadin for 6 weeks for nocturia in patients with multiple sclerosis reported more side effects during the placebo arm than during melatonin but circulating melatonin levels were not measured (23).

Previous studies investigating melatonin administration during real-life shift working in healthcare workers have used various doses of either immediate or modified release melatonin: 1mg (24), 5 mg (25) or 6mg (26). All of these studies had a similar trial design to our study and the intervention was given after the nightshift, immediately before participants went to bed, i.e., in the morning. Modified release Circadin is currently licensed in the EU for treatment of primary insomnia at a dose of 2 mg. We chose to use 6 mg Circadin since this dose had been shown to maximize sleep-promoting effects while minimizing detrimental effects on functional performance in military aircrew given a single dose (27). Our previous work showed no concerning effects even after 100 mg of immediate release melatonin (18).

Nightshift working in our study affected sleep duration, WASO and propensity for situational sleepiness measured using ESS. Sleep duration determined by Actigraphy decreased markedly across the shifts, regardless of treatment arm, and indicates the complexity of disentangling the temporal effects of shift work on sleep. What is of particular concern is that some subjects slept less than 2 h before going back to work again in a busy clinical environment. We are aware that some participants will have had domestic/caring duties to attend to which may have compromised the amount of time available to them for daytime sleeping after their shifts had ended. Sleep disturbance, measured here using the VSH sleep scale, significantly improved during each series of shifts, notably so after shift 3, and, although there was no significant effect of Circadin overall, there was less sleep disturbance after shift 3 during melatonin treatment. Actigraphy is much more convenient compared to polysomnography but periods of inactivity may be recognize as “sleep” such that latency may be underestimated and duration overestimated (17). However in a cross over trial in healthy subjects the impact of this is minimal. Sleep latency (the time taken to fall asleep) measured by Actigraphy, appeared faster after the first shift than subsequent shifts, which may be expected given most subjects had usually not slept for around 24 h at this point. We found no significant effect of melatonin treatment on latency, in concurrence with previous studies of real-life nightshifts using immediate release melatonin (24–26). Studies in patients with pre-existing sleep issues have reported variable effects of melatonin on latency (23).

Self-reported sleepiness is acknowledged as a sensitive indicator of insufficient sleep and both the ESS and other similar scores are widely used (15, 25, 28–30). ESS has not been specifically validated for changes in individuals over time, but more sleepiness during and after shifts than before was very apparent here, with some improvement by the third shift, suggesting adaptation. No interventional study of real-life shift working has reported sleepiness scores during shifts. We found a small but statistically significant effect of melatonin treatment on ESS during the second shift. The reason for this is not clear and this did not impact on performance in attention/concentration tasks.

DDAT assesses attention and concentration, not working memory or mathematical ability; the level is that of a child aged around 8 years (31). It is said not to be affected by learning although we found that scores improved over both series of shifts perhaps suggesting otherwise, but with a significant treatment effect on DDAT beyond any adaptation or practice effects. There was no capacity for participants to learn the actual answers to the sums as these were completely different each time. However the technique of undertaking the sums can be learned much like a child learning what to do to get the answer rather than learning the actual answer to a particular sum. A previous study of ICU nurses found adverse effects of nightshifts on mathematical problem-solving (15). No other studies have investigated the effect of melatonin treatment on DDAT.

The main finding in relation to PVT was pronounced differences between male and female participants, which we have noted previously in members of the public. It is worth stating that binary assignment to “male” or “female” was assumed based on participants’ names and appearance. Anecdotally PVT has been reported to be ‘virtually unaffected’ by aptitude or learning (14, 32), although more recent work disputes this (33). PVT response is impaired in sleep-deprived medical interns (34) but sex differences in performance were not reported. Some studies have described sex differences (35, 36); some have included only one sex, but most do not consider the effect of sex at all. Reporting the sex of the participants in randomized studies using PVT as a functional assessment is clearly important. We found that PVT reaction times were worse after each shift than before in all participants regardless of treatment arm.

In summary, fatigue and sleep deprivation which could result in lapses of vigilance or judgement while doctors or nurses are working nightshifts, might impact on patient safety; melatonin administration may benefit sleep and functional performance during nightshift working. This pilot study shows that administration of melatonin as Circadin to clinical staff working nightshifts is safe with no concerning side effects and the trial design was feasible, and acceptable to participants, with no wellbeing concerns raised. Only one person said they would not take melatonin while working nightshifts if it was available. The wearing of the actigraphy watch for objective assessment of sleep parameters was well tolerated and did not interfere with normal activity. No previous studies have undertaken such a comprehensive and robust study of administration of melatonin during real-life nightshift working to assess safety, feasibility and acceptability along with an exploration of effects on sleep and functional capacity.

Other medications are available which may be of help to those working nightshifts. Armodafinil and Modafinil are similar to amphetamines and promote wakefulness through an unknown mechanism. Armodafinil can be addictive with a number of unpleasant side effects and Modafinil has drug interactions (for example with cyclosporin, phenytoin and the contraceptive pill). Ramelteon and tasimelteon are melatonin receptor agonists; the former is licensed for sleep latency issues and the latter for non-24 h sleep-wake disorder in blind individuals. Neither drug has been tested in the context of nightshift working. Melatonin, in comparison, is without concerning side effects.

Although there were some significant findings in our study this was a pilot feasibility study and was designed to test the feasibility and acceptability of the trial design. A type II error (insufficient power) may mean that our inability to find an effect may not necessarily mean there was no effect. A properly powered definitive trial is the next step. We conclude that this trial design for a larger definitive study is both feasible and safe.

## Data Availability Statement

The raw data supporting the conclusions of this article will be made available by the authors, without undue reservation.

## Ethics Statement

The studies involving human participants were reviewed and approved by North of Scotland Research Ethics Service, reference 16/NS/0010. The patients/participants provided their written informed consent to participate in this study.

## Author Contributions

HG was Chief Investigator and drafted the manuscript. HG and NW conceived of and designed the study and contributed to data analysis and interpretation. BT had responsibility for overall trial management, and contributed to participant recruitment, sample collection, and data acquisition. LA contributed to recruitment and data acquisition. MC undertook data analysis. All authors contributed to the article and approved the submitted version.

## Funding

The study was funded by the British Journal of Anaesthesia and the Royal College of Anaesthetists, funding partners of the National Institute of Academic Anaesthesia (grant number WKR0-2015-0027).

## Conflict of Interest

The authors declare that the research was conducted in the absence of any commercial or financial relationships that could be construed as a potential conflict of interest.
