# Supplementary material for: Melatonin Is a Feasible, Safe, and Acceptable Intervention in Doctors and Nurses Working Nightshifts: The MIDNIGHT Trial
Source: Front Psychiatry. 2020 Aug 27;11:872. doi: 10.3389/fpsyt.2020.00872 (PMC7481467; doi:10.3389/fpsyt.2020.00872)
Supplement: Supplementary file 1 [file Table_1.docx]

**Melatonin is a feasible, safe and acceptable intervention in doctors and nurses working nightshifts: the MIDNIGHT trial**

Bensita MVJ Thottakam^1^, Nigel R Webster^1,2^, Lee Allen^2^, Malachy O Columb^3^ and Helen F Galley^1,2*^

^1^Institute of Medical Sciences, University of Aberdeen, Aberdeen; ^2^Intensive Care Unit, Aberdeen Royal Infirmary, Aberdeen and ^3^ Manchester University Hospitals NHS Foundation Trust, Wythenshawe, UK.

* Corresponding author Helen Galley. Institute of Medical Sciences, University of Aberdeen, Aberdeen AB25 2ZD, UK. Email [h.f.galley@abdn.ac.uk](mailto:h.f.galley@abdn.ac.uk)

**Supplementary data: Biochemistry and haematology results**

|  | Placebo | | Melatonin | | Treatment  *P* value | Time  *P* value | Period  *P* value | Sequence  *P* value |
| --- | --- | --- | --- | --- | --- | --- | --- | --- |
|  | Start | End | Start | End |  |  |  |  |
| Na^+^ (mmol/L) | 141.0  (1.7) | 141.5  (1.6) | 141.4  (1.9) | 141.3  (1.4) | 0.75 | 0.40 | 0.85 | 0.68 |
| K^+^ (mmol/L) | 3.73  (0.28) | 3.73  (0.29) | 3.67  (0.31) | 3.70  (0.29) | 0.50 | 0.70 | 0.01 | 0.79 |
| Cl^-^ (mmol/L) | 104.8  (2.2) | 105.8  (2.2) | 104.4  (2.9) | 105.7  (2.53) | 0.46 | 0.013 | 0.57 | 0.86 |
| Urea (mmol/L) | 4.22  (0.99) | 4.21  (1.05) | 4.32  (1.08) | 4.30  (1.05) | 0.52 | 0.94 | 0.049 | 0.88 |
| Creatinine (µmol/L) | 68.8  (12.1) | 66.7  (10.2) | 67.8  (10.9) | 66.6  (12.2) | 0.92 | 0.43 | 0.0056 | 0.91 |
| Albumin (g/L) | 43.4  (3.0) | 43.2  (2.3) | 43.4  (3.2) | 42.3  (3.7) | 0.71 | 0.33 | 0.16 | 0.71 |
| Bilirubin (µmol/L) | 11.2  (5.2) | 12.3  (6.0) | 10.2  (4.5) | 10.7  (3.9) | 0.059 | 0.28 | 0.0074 | 0.90 |
| ALP (IU/L) | 68.4  (15.5) | 67.0  (17.4) | 67.8  (15.0) | 66.5  (15.4) | 0.75 | 0.55 | 0.0001 | 0.68 |
| ALT (IU/L) | 15.0  [11.0-33.0] | 13.5  [11.3-28.0] | 17.0  [12.5-31.5] | 18.5  [11.0-34.8] | 0.48 | 0.66 | 0.21 | 0.75 |
| γGT (iu L^-1^) | 16.0  [12.5-23.0] | 15.0  [12.0-20.8] | 17.0  [11.0-23.5] | 14.5  [10.3-23.8] | 0.85 | 0.99 | 0.18 | 0.66 |
| Hb (g/L) | 139.0  (11.5) | 134.7  (10.5) | 138.8  (13.1) | 134.3  (12.14) | 0.84 | 0.042 | 0.60 | 0.84 |
| WCC (x10^9^/L) | 7.12  (1.59) | 6.07  (1.48) | 7.01  (1.77) | 5.85  (1.52) | 0.46 | 0.0001 | 0.15 | 0.70 |

Data are mean (SD) or median [interquartiles]. Alkaline phosphatase (ALP), alanine transaminase (ALT); γ-glutamyl transferase (γGT), haemoglobin (Hb), leucocyte count (WCC).
